# The definition of remission in asthma should be unique and specific

**DOI:** 10.5578/tt.202402909

**Published:** 2024-06-12

**Authors:** İnsu YILMAZ, Gülden PAÇACI ÇETİN, Murat TÜRK

**Affiliations:** 1 Division of Immunology and Allergy, Department of Chest Diseases, Erciyes University Faculty of Medicine, Kayseri, Türkiye; 2 Clinic of Immunology and Allergy, Kocaeli City Hospital, Kocaeli, Türkiye

Remission, a phrase more commonly used with rheumatologic diseases and malignancies, is defined as the lessening of the severity of a disease or symptom; disappearance of symptoms or cessation of the activity of a disease for a period (1). Following recent advances in the treatment of the disease, the term remission has been widely and increasingly used for asthma. In the literature, there exist several different definitions of remission; such as “remission on treatment”, “remission off treatment”, “complete remission on treatment”, “complete remission off treatment”. Studies published before 2020 defined asthma remission as a symptom-free state lasting from six months to three years. Furthermore, definitions of clinical remission were based on patient-reported symptoms and treatment. In contrast, recent studies have determined more clear and objective criteria for the definition of remission in asthma (Table 1) (2-4). Although the new criteria are stricter and understandable, we believe that the existence of more than one remission concept and defining definitions of new remission concepts daily such as “inflammatory remission” or “deep remission” are confusing in clinical practice. We strongly believe that the definition of remission should not be confusing, should be clean, very specific and cover a single definition as: “Complete remission of asthma without any controller treatment”. Remission in asthma should define a childhood asthma that might goes into clinical, functional and inflammation-free state without any regular controller treatment (off treatment). It does not reflect a definite cure, instead the disease may reappear later. In adults, it also means that the disease can be silent when the above complete remission off therapy is met, but it does not mean that asthma will not occur again in the future.

**Table:** 

**Table 1.** Studies defining asthma remission criteria				
** Clinical remission (all criteria, at least 12 months) **		Menzies-Gow et al., 2020, (2)	** Complete remission (all criteria, at least 12 months) **	
**On treatment**	**Off treatment**		**On treatment**	**Off treatment**
No significant asthma symptoms Normalization/stabilization of lung function The patient agrees that the disease is in remission Not using systemic corticosteroids for either exacerbations or long-term control of disease	Not receiving any treatment for asthma for at least 12 No significant asthma symptoms Normalization/stabilization of lung function The patient agrees that the disease is in remission Not using systemic corticosteroids for either exacerbations or long-term control of disease		No significant asthma symptoms Normalization/stabilization of lung function The patient agrees that the disease is in remission Not using systemic corticosteroids for either exacerbations or long-term control of disease and Objective proof that the inflammation associated with asthma that was previously documented has resolved (e.g., decreased sputum or blood eosinophil counts, FENO, etc. ), and if suitable, negative bronchial hyperresponsiveness	Not receiving any treatment for asthma for at least 12 No significant asthma symptoms normalization/stabilization of lung function The patient agrees that the disease is in remission Not using systemic corticosteroids for either exacerbations or long- term control of disease and Objective proof that the inflammation associated with asthma that was previously documented has resolved (e.g., decreased sputum or blood eosinophil counts, FENO, etc. ), and if suitable, negative bronchial hyperresponsiveness
Clinical remission either on or off treatment (all criteria, at least 12 months)		Thomas D. et al., 2022, (3)	Complete remission either on or off treatment (all criteria, at least 12 months)	
No symptoms No exacerbations Optimization of lung function			No symptoms No exacerbations Optimization of lung function Normalization or stability of any underlying pathology	
Clinical remission on treatment (all criteria, at least 12 months)		Blaiss et al., 2023, (4)		
No exacerbations No interruptions from school or work due to symptoms of asthma Results for pulmonary function that are consistently stable and optimal, with at least two assessments made over the year ICS, ICS/LABA, and leukotriene receptor antagonist controller treatments should be used, but only at low-to-medium doses ACT> 20, AirQ< 2, ACQ< 0.75 during the whole 12-month period assessed, with ≥2 measures throughout the year Symptoms requiring one-time reliever therapy no more than once a month				
ICS: Inhaled corticosteroid, LABA: Long acting beta agonist, ACT: Asthma control test, AirQ: Asthma impairment and risk questionnaire, ACQ: Asthma control questionnair.

Remission in asthma

We recognize that the definition of asthma remission, if defined as we propose, would have very strict criteria. However, it would be more accurate to express real remission this way. According to Wikipedia; in cancer-treatment, doctors usually avoid the term “cured” and instead prefer the term “no evidence of disease” to refer to a complete remission of cancer, which does not rule out the possibility of relapse (5,6). In asthma, the concept of remission can be used to mean the absence of evidence of disease. As with cancer, this does not mean that there will be no relapse.

Another issue we want to focus attention on is that we already have definitions such as asthma control (according to ACT or ACQ and no attacks in the last year) and symptom control expressed as full control, partial control or uncontrolled. In addition, terms such as super responder, responder, and non- responder, which determine the response to treatment, are already used. While there is still no consensus even in the criteria of super-responder, the use of the definition of remission on treatment overlapping with the super-responder terminology may cause more confusion.

In conclusion, while there are terminologies that define asthma control and express complete control, various concepts of remission will only cause confusion. In particular, the concept of “remission on treatment” overlapping with existing definition (super-responder) should not be used to avoid confusion. We think that if the concept of remission is to be used in asthma, a single remission concept

should be used, and this should be defined in accordance with the “complete remission off treatment” criteria. However, even after complete remission, it should be recognized that the disease may relapse at some point.

## REFERENCES

1 *
  Oxford English Dictionary. Available from: https://wwwoed.com/search/dictionary/?scope=Entries&q=remission (Accessed Date: 27 March 2024)..
  *
2 *
  Menzies-Gow A, Bafadhel M, Busse WW, Casale TB, Kocks JWH, Pavord ID, et al. An expert consensus frame- work for asthma remission as a treatment goal. J Allergy Clin Immunol 2020; 145(3): 757-65. https://doi.org/10.1016/j.jaci.2019.12.006*
3 *
  Thomas D, McDonald VM, Pavord ID, Gibson PG. Asthma remission: What is it and how can it be achieved? Eur Respir J 2022; 60(5): 2102583. https://doi.org/10.1183/13993003.02583-2021*
4 *
  Blaiss M, Oppenheimer J, Corbett M, Bacharier L, Bernstein J, Carr T, et al. Consensus of an American College of Allergy, Asthma, and Immunology, American Academy of Allergy, Asthma, and Immunology, and American Thoracic Society workgroup on definition of clinical remission in asthma on treatment. Ann Allergy Asthma Immunol 2023; 131(6): 782-5. https://doi.org/10.1016/j.anai.2023.08.609*
5 *
  What does cancer remission, recurrence & cure mean? Accessed date: 2024 April 4. Available from: http://www.mskcc.org/experience/living-beyond-cancer/survi-vorship/what-cure*
6 *
  Remission: What does it mean? Accessed date: 2024 April 4. Available from: https://web.archive.org/web/20190406074413/https://www.webmd.com/cancer/remission-what-does-it-mean*

